# Long non-coding RNA LUCAT1 is associated with poor prognosis in human non-small cell lung cancer and regulates cell proliferation via epigenetically repressing p21 and p57 expression

**DOI:** 10.18632/oncotarget.16044

**Published:** 2017-03-09

**Authors:** Yue Sun, Shi-Dai Jin, Quan Zhu, Liang Han, Jing Feng, Xi-Yi Lu, Wei Wang, Feng Wang, Ren-Hua Guo

**Affiliations:** ^1^ Department of Oncology, Yancheng Third People's Hospital, The Affiliated Yanchen Hospital of Southeast University Medicine College, Yancheng, Jiangsu, China; ^2^ Department of Oncology, First Affiliated Hospital of Nanjing Medical University, Nanjing, Jiangsu, China; ^3^ Department of Thoracic Surgery, First Affiliated Hospital of Nanjing Medical University, Nanjing, Jiangsu, China; ^4^ Department of Oncology, Xuzhou Central Hospital, Affiliated Xuzhou Hospital, College of Medicine, Southeast University, Xuzhou, Jiangsu, China; ^5^ Department of Oncology, Jiangsu Shengze Hospital, Suzhou, Jiangsu, China; ^6^ Department of Oncology, Yancheng Third People's Hospital, The Affiliated Yanchen Hospital of Southeast University Medicine College, Yancheng, Jiangsu, China

**Keywords:** LncRNAs, LUCAT1, p21, p57, non-small cell lung cancer (NSCLC)

## Abstract

Recently, long non-coding RNAs (lncRNAs) have been recognized as playing key roles in regulating cellular processes, such as proliferation, invasion, and metastasis. These lncRNAs have been shown to be abnormally expressed in tumorigenic processes. However, the role and clinical relevance of LUCAT1 in non-small-cell lung cancer (NSCLC) remain unclear. In this study, we found that the expression of LUCAT1 was significantly up-regulated in NSCLC tissues compared to non-tumor tissues, and its expression was associated with tumor size, tumor–node–metastasis (TNM) stage and overall survival (OS). Further experiments showed that LUCAT1 knockdown inhibited cell proliferation both *in vitro* and *in vivo*. Mechanistic investigations showed that LUCAT1 plays a key role in G0/G1 arrest. We further demonstrated that LUCAT1 was associated with polycomb repressor complexes (PRC2) and that this association was required for epigenetically repression of p21 and p57, thus contributing to the regulation of NSCLC cell cycle and proliferation. In summary, our results show that LUCAT1 could regulate tumorigenesis of NSCLC and be biomarker for poor prognosis in NSCLC.

## INTRODUCTION

Lung cancer is the main cause of cancer death world-wide, and NSCLC accounts for nearly 85% of those cases [1]. And although clinical and experimental oncology have been exceptional progress in recent years, the prognosis for the majority of lung cancer cases remains poor, as the 5-year overall survival rate is approximately 15% [2, 3]. Therefore, there is an urgent need to provide detailed knowledge of the molecular mechanisms underlying NSCLC progression, which is crucial for improving the prevention, diagnosis, treatment, and survival rate of NSCLC.

Recently, with the development of molecular genetics and molecular biology, non-coding RNAs have risen to the forefront of cancer research. Recent reports have shown that non-coding RNAs (ncRNAs), which have been classified as small ncRNAs (sncRNAs; less than 200 nucleotides) and long non-coding RNAs (lncRNAs; greater than 200 nucleotides) [4], are transcribed from approximately 90% of the genomic DNA [5, 6]. A large number of studies have revealed that aberrant expression of lncRNAs can influence diverse cellular and oncogenic processes [7, 8]. The dysregulation of lncRNAs has a functional role in diverse cancers, such as lung cancer, gastric cancer, and breast cancer [9–11]. In addition, researchers have also reported that lncRNAs could serve as biomarkers for the diagnosis of a variety of diseases [12, 13]. Hence, it is important to identify cancer-associated lncRNAs and investigate their molecular and biological roles in tumors.

Several research studies have revealed that lncRNAs play significant roles in recruiting PRC2 [14], which contain histone methyltransferase. Histone methyltransferase primarily induces the trimethylation of histone H3 lysine 27 on target genes [15, 16]. Previous studies have shown that the dysregulated expression of EZH2, one of the three core components of PRC2 (EZH2, SUZ12, and EED), could be a biomarker in diverse cancers, such as lung cancer, gastric cancer, hepatocellular carcinoma and cervical cancer [17–20].

In this study, we investigate lung cancer associated transcript 1 (LUCAT1), which is found in the airway epithelium of cigarette smokers. LUCAT1 is located on chromosome 5 [21]. Thus far, the functions and molecular mechanism of LUCAT1 in NSCLC remain unclear. Therefore, we were prompted to explore the role of LUCAT1 in human NSCLC. In our study, we discovered that lncRNA LUCAT1 was significantly increased in NSCLC tissues compared with the adjacent non-cancerous tissues. In addition, LUCAT1 knockdown repressed NSCLC proliferation both *in vitro* and *in vivo*. RNA-binding protein and chromatin immunoprecipitation (RIP and ChIP) assays revealed that LUCAT1 epigenetically repressed the expression of p21 and p57 via associating with PRC2. These data support a role for LUCAT1 in regulating both the NSCLC cell cycle and proliferation.

## RESULTS

### LUCAT1 expression is increased in human non-small lung cancer (NSCLC) and is associated with poor prognosis

**Figure 1:**
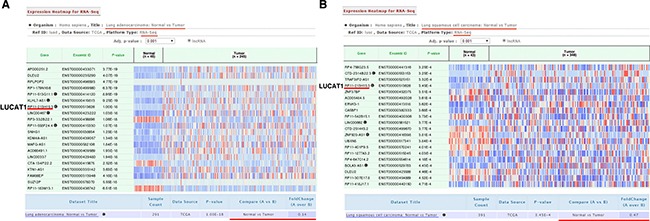
Selecting out LUCAT1 through bioinformatics analysis (**A**) And (**B**) The bioinformatics tools “lncRNAtor” (http://lncrnator.ewha.ac.kr/index.htm) was used to analyze the RNA-Seq data from TCGA of lncRNA of lung adenocarcinoma and lung squamous cell carcinoma. Lung adenocarcinoma: Normal (*N* = 46), Tumor (*N* = 245). Lung squamous cell carcinoma: Normal (*N* = 43), Tumor (*N* = 348).

**Figure 2:**
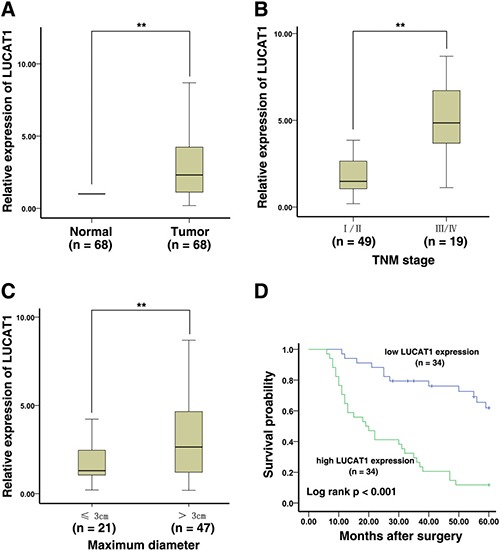
LUCAT1 expression is increased in NSCLC and is associated with poor prognosis (**A**) The LUCAT1 expression in NSCLC tissues (*n* = 68) increased significantly compare to the corresponding non-tumor tissues (*n* = 68). LUCAT1 expression was detected by qPCR and normalized to GAPDH expression. (**B**) And (**C**) Higher LUCAT1 expression was positively associated with lager tumor size and advanced TNM stage. (**D**) The overall survival was analyzed by Kaplan-Meier analysis, according to LUCAT1 expression levels. *P < 0.05, **P < 0.01.

First, we used the bioinformatics tools “lncRNAtor” (http://lncrnator.ewha.ac.kr/index.htm) to analyze RNA-Seq data from TCGA (The Cancer Genome Atlas) of lncRNAs of lung squamous cell carcinoma (*N* = 391) and lung adenocarcinoma (*N* = 291). The results showed that the average fold change (normal vs tumor) was 2.31 in lung squamous cell carcinoma and 2.99 in lung adenocarcinoma (Figure 1A and 1B). Then, we investigated whether the expression of LUCAT1 was altered in NSCLC tissues, we performed qRT-PCR in 68 patients with NSCLC. The results revealed that the level of LUCAT1 increased significantly in the corresponding adjacent non-normal tissues; as shown in Figure 2A, the LUCAT1 level in NSCLC tissues were nearly 3.79-fold enhanced compared with normal controls. Subsequently, we assessed the correlation between LUCAT1 expression and the clinicopathological features in patients with NSCLC. According to the collected clinical information, there was a statistically significant association between increased LUCAT1 levels and larger tumor size, and advanced TNM stage (Figure 2B and 2C). However, several other clinical parameters, such as gender (male, female), histological grade (low or undifferentiated, moderate, or high), lymphatic metastasis (N0, N1, or above), and distant metastasis (M0, M1), showed no obvious correlation with LUCAT1 expression in our study (Table 1).

**Table 1:** The relationship between LUCAT1 expression and clinicpathological factors of 68 NSCLC patients

Characteristics	Expression of LUCAT1		*p* value*
	low	high	
Sex			0.31
male	24	20	
female	10	14	
Age			0.028*
≤ 60	14	23	
> 60	20	11	
Histological grade			0.476
Middle or low	28	31	
high	6	3	
Histological classification			0.324
SCC (Squamous cell carcinoma)	22	18	
AD (adenocarcinoma or other)	12	16	
TNM stage			< 0.001**
I and II	31	18	
III and IV	3	16	
Lymph node metastasis			0.22
negative	17	12	
positive	17	22	
Tumor size			0.018*
≤ 3 cm	15	6	
>3 cm	19	28	
History of smoking			0.618
Ever	20	22	
Never	14	12	
Chi-square test			

^*^p < 0.05.

^**^p < 0.01.

At the same time, we were also interested in examining the relationship between LUCAT1 expression and the prognosis of patients with NSCLC. Specifically, we explored the association between LUCAT1 expression and clinical outcomes and overall survival. We divided the patients into two groups on the basis of the median expression for LUCAT1 in tumor tissues: LUCAT1-high group (above the median, *n* = 34) and LUCAT1-low group (below the median, *n* = 34). We then performed a Kaplan-Meier survival analysis and log-rank tests. The results of these analyses showed that the LUCAT1-high group has statistically poorer overall survival (*P* < 0.001) (Figure 2D)than the LUCAT1-low group. In addition, the univariate and multivariate survival analyses (Cox proportional hazards regression model) were used to further assess whether LUCAT1 expression can be a novel prognostic or progression marker for NSCLC patients. Univariate analysis showed that a decreased OS was related to TNM stage (I/II, III/IV) (*P* < 0.001) and LUCAT1 expression (*P* < 0.001). Multivariate analysis revealed that LUCAT1 expression could be an independent prognostic indicator for OS in patients with NSCLC (Table 2).

**Table 2:** Univariate and multivariate analysis of clinic pathologic factors for overall survival in 68 patients with NSCLC

Risk factors	Univariate analysis			multivariate analysis		
	HR*	*p* value	95% CI	HR	*p* value	95% CI
LUCAT1 expression	1.073	< 0.001**	1.040~1.108	1.085	< 0.001**	1.045~1.126
TNM stage (I/II, III/IV)	5.796	< 0.001**	2.977~11.281	5.7	< 0.001**	2.917~11.136
Tumor size (≤ 3 cm, > 3 cm)	1.657	0.163	0.814~3.373			
Histological grade (middle or low, high)	0.92	0.85	0.387~2.187			
Histological classification (SCC, AD or another)	1.145	0.664	0.621~2.111			
Age (≤ 60, >60)	0.648	0.168	0.349~1.201			
N (negative, positive)	1.712	0.097	0.907~3.234			
History of smoking (ever, never)	1.512	0.183	0.823~2.779			
Sex (male, female)	1.323	0.402	0.687~2.549			
*HR hazard ratio						

^*^*p < 0.01.

### Silencing of LUCAT1 inhibits NSCLC cell growth and induces cell apoptosis *in vitro*

**Figure 3:**
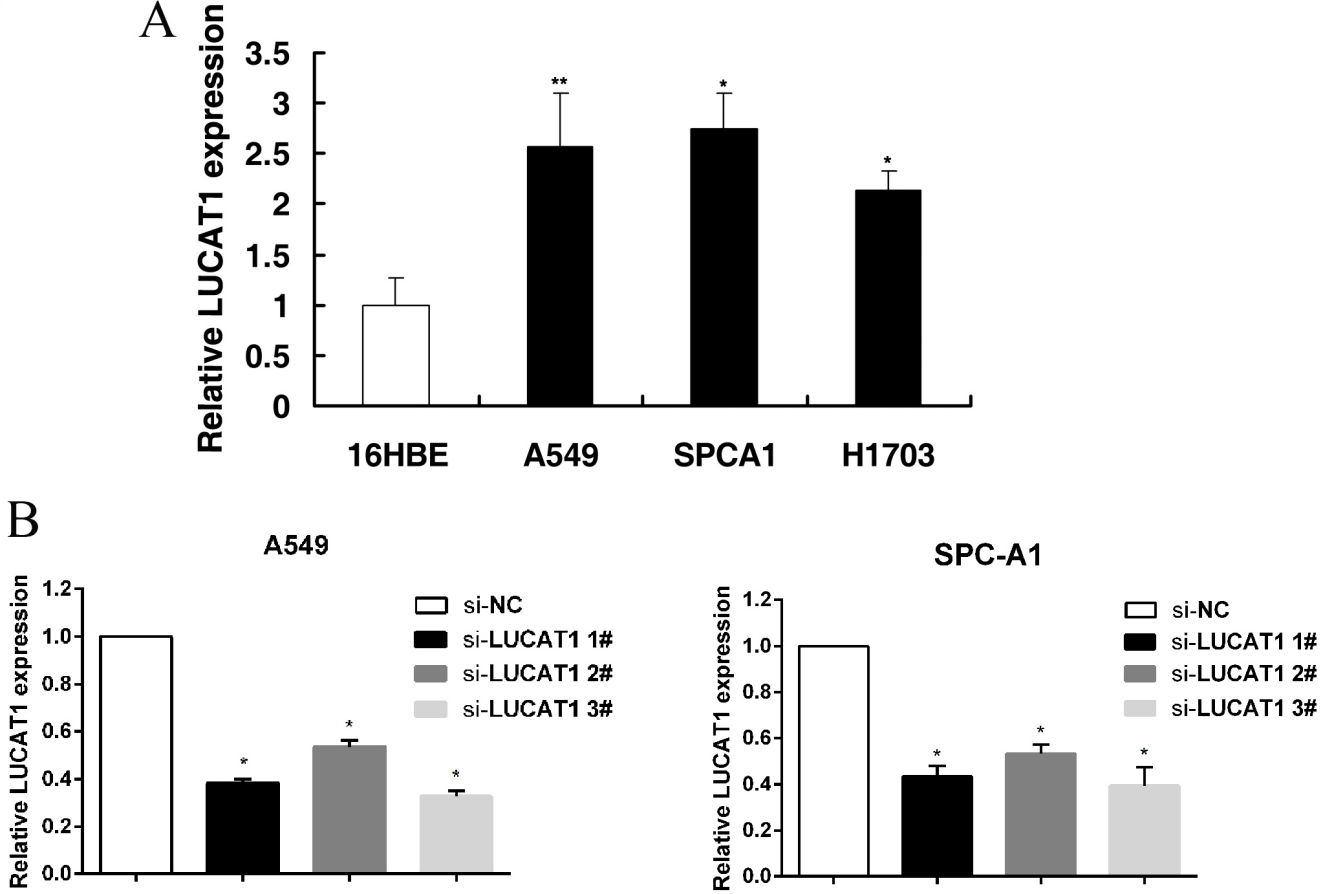
The expression of LUCAT1 in NSCLC cell lines (**A**) The three NSCLC cell lines (A549, SPC-A1, and H1703) expressed higher levels of LUCAT1 than the normal bronchial epithelial cell line (HBE). (**B**) At 48 h after transfection, the LUCAT1 expression was analyzed by qRT-PCR. *P < 0.05, **P < 0.01

To further understand the potential role of LUCAT1 in NSCLC, qRT-PCR analysis was used to detect the expression of LUCAT1 in three NSCLC cell lines: A549, SPC-A1 and H1703. As shown in Figure 3A, the three cell lines expressed higher levels of LUCAT1 than the normal bronchial epithelial cell line (HBE). The cell lines with the higher relative expression (A549 and SPC-A1) were chosen for further study. LUCAT1 was silenced in NSCLC cells by transfection of LUCAT1 siRNA into A549 and SPC-A1 cell lines. After 48 h post-transfection, qRT-PCR analysis showed that LUCAT1 expression was clearly reduced (Figure 3B). This analysis also revealed that si-LUCAT1 1# and si-LUCAT1 3# had higher efficiency of interference than si-LUCAT1 2#, so we chose si-LUCAT1 1# and si-LUCAT1 3# for further experiments.

**Figure 4:**
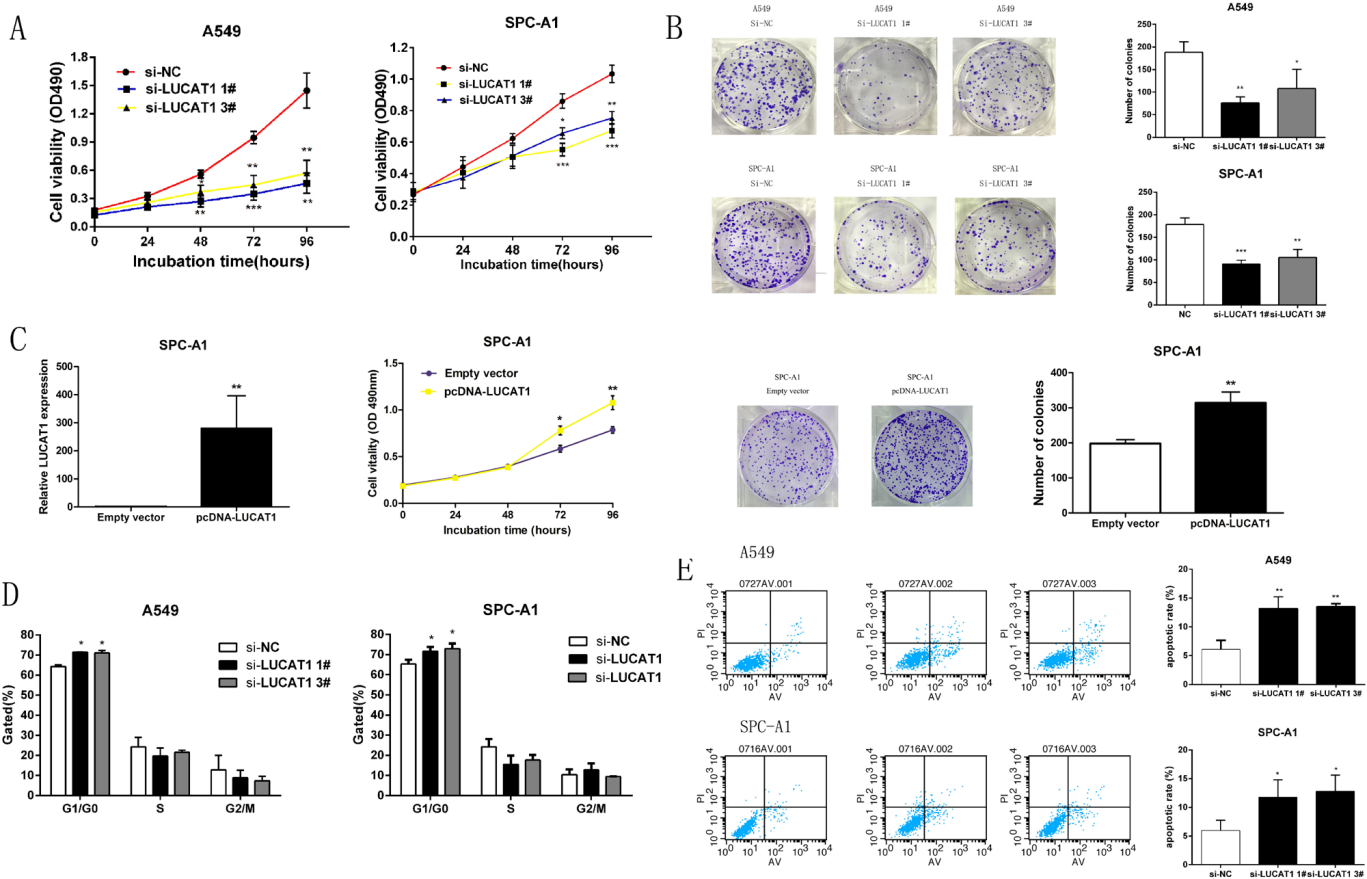
LUCAT1 regulates NSCLC cell growth *in vitro* (**A**) At 48 h after transfection, MTT assay was performed to detect the proliferation of A549 and SPC-A1 cells. (**B**) The results of colony formation of A549 and SPC-A1 cells transfected with si-RNA against LUCAT1. (**C**) MTT and colony formation assays were performed to determine cell proliferation of SPCA1 cells after transfection of overexpression plasmid LUCAT1. (**D**) At 48 h after transfection, cell cycle was analyzed by flow cytometry. The bar chart represented the percentage of cells in GO/G1, S, or G2/M phase, as indicated. (**E**) At 48 h after transfection, the apoptotic rates of cells were detected by flow cytometry. LR, early apoptotic cells. UR, terminal apoptotic cells. Error bars indicate means ± S.E.M. *P < 0.05, **P < 0.01, ***P < 0.001.

To explore the effect of LUCAT1 on NSCLC cell growth, cell proliferation was meaured by MTT assays. As shown in Figure 4A, knockdown of LUCAT1 expression markedly inhibited cell proliferation compared to the control cells. Colony-formation assays also revealed that clonogenic survival was significantly decreased following the inhibition of LUCAT1 both in A549 and SPC-A1 cell lines (Figure 4B). In contrast, overexpressed LUCAT1 could promote cell proliferation. Similarly, the result of colony-formation assay revealed that overexpression of LUCAT1 could boost the number of clones (Figure 4C).

The effect of LUCAT1 on cell cycle progression was then examined using flow cytometry. The results revealed that, compared to the scrambled siRNA control transfected cells, the cell-cycle progression of si-LUCAT1 cells was significantly arrested in G0/G1 phase both in A549 and SPC-A1 cell lines (Figure 4D). In addition, the results of flow-cytometric analysis of apoptosis showed that LUCAT1 knockdown increased the proportion of apoptotic cells (Figure 4E). In summary, these data suggest that LUCAT1 plays a critical role in human NSCLC and that knockdown of LUCAT1 inhibits cell proliferation and induces apoptosis in NSCLC cells.

### Knockdown LUCAT1 inhibits NSCLC cell proliferation *in vivo*

**Figure 5:**
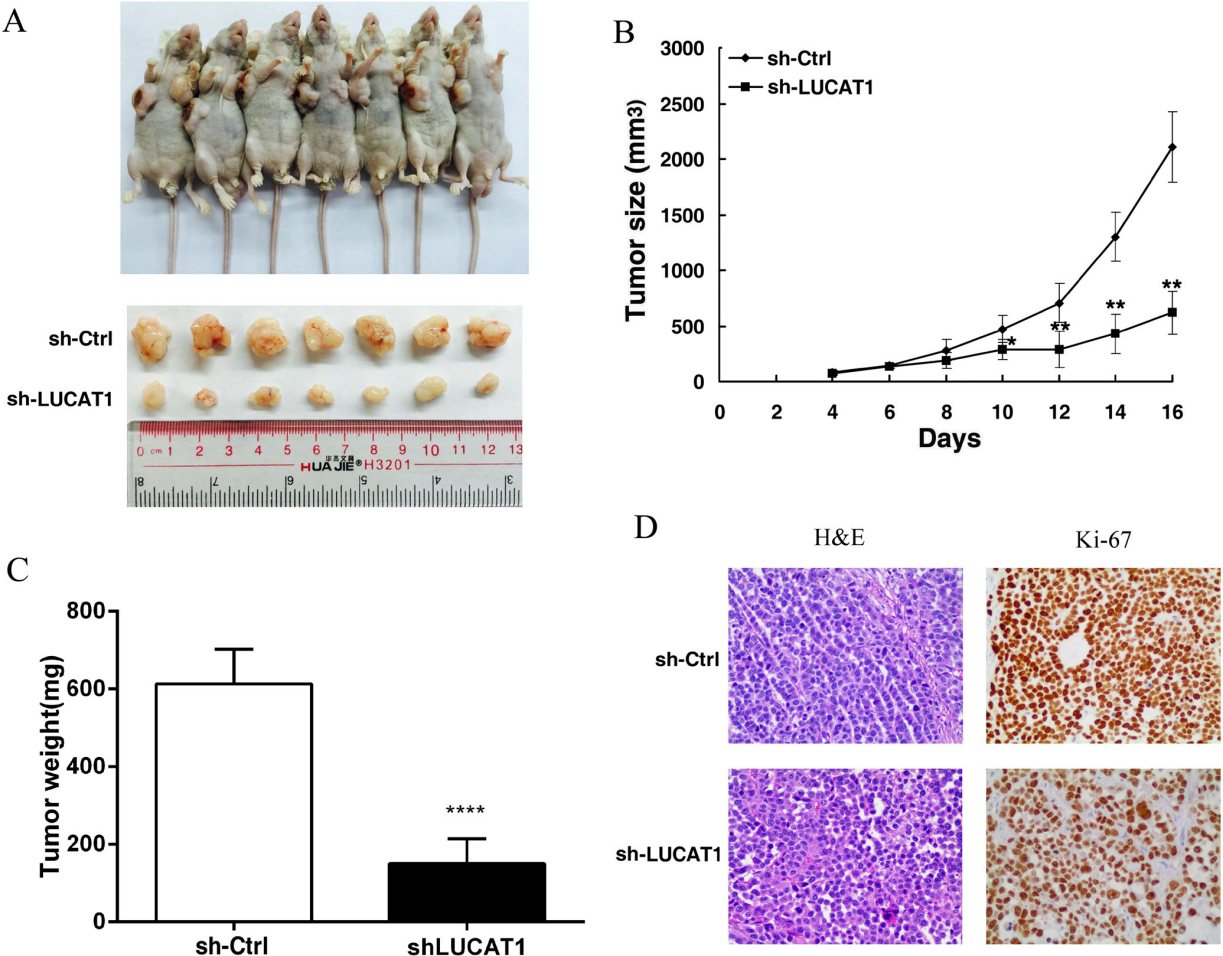
The impact of LUCAT1 on tumorigenesis *in vivo* (**A**) And (**B**) Scramble or shLUCAT1 was transfected into SPC-A1 cells, which were injected into nude mice (*n* = 7). The tumor volumes were calculated every 2 days after injection. The bars indicate SD. (**C**) The tumor weights are shown as means of tumor weights ± S.D. (**D**) Histopathology of xenograft tumors. The tumor sections underwent H&E staining and IHC staining using antibodies against Ki-67. Error bars indicate means ± S.E.M. *P < 0.05, **P < 0.01. ***P < 0.001.

To determine the effects of LUCAT1 on tumorigenesis *in vivo*, we investigated the effect of LUCAT1 knockdown in nude mice harboring NSCLC xenografts. We subcutaneously injected SPC-A1 cells transfected with either sh-LUCAT1 or empty vector into flanks of 5 week-old nude mice. By 16 days after injection, all mice developed xenograft tumors at the injection site. We found that tumor growth in the sh-LUCAT1 group was measurably slower than that in the empty vector group (Figure 5A and 5B). Moreover, the average tumor weight was distinctly lower in the sh-LUCAT1 group compared with the control group (Figure 5C). Additionally, we found that the tumors that developed from cells transfected with sh-LUCAT1 displayed lower Ki-67 expression compared to tumors formed from cells transfected with empty vector (Figure 5D). These results provide further evidence that down-regulated expression of LUCAT1 is significantly correlated with the decreased proliferative capacity of NSCLC cells.

### Evidence of association between PRC2 and LUCAT1

**Figure 6:**
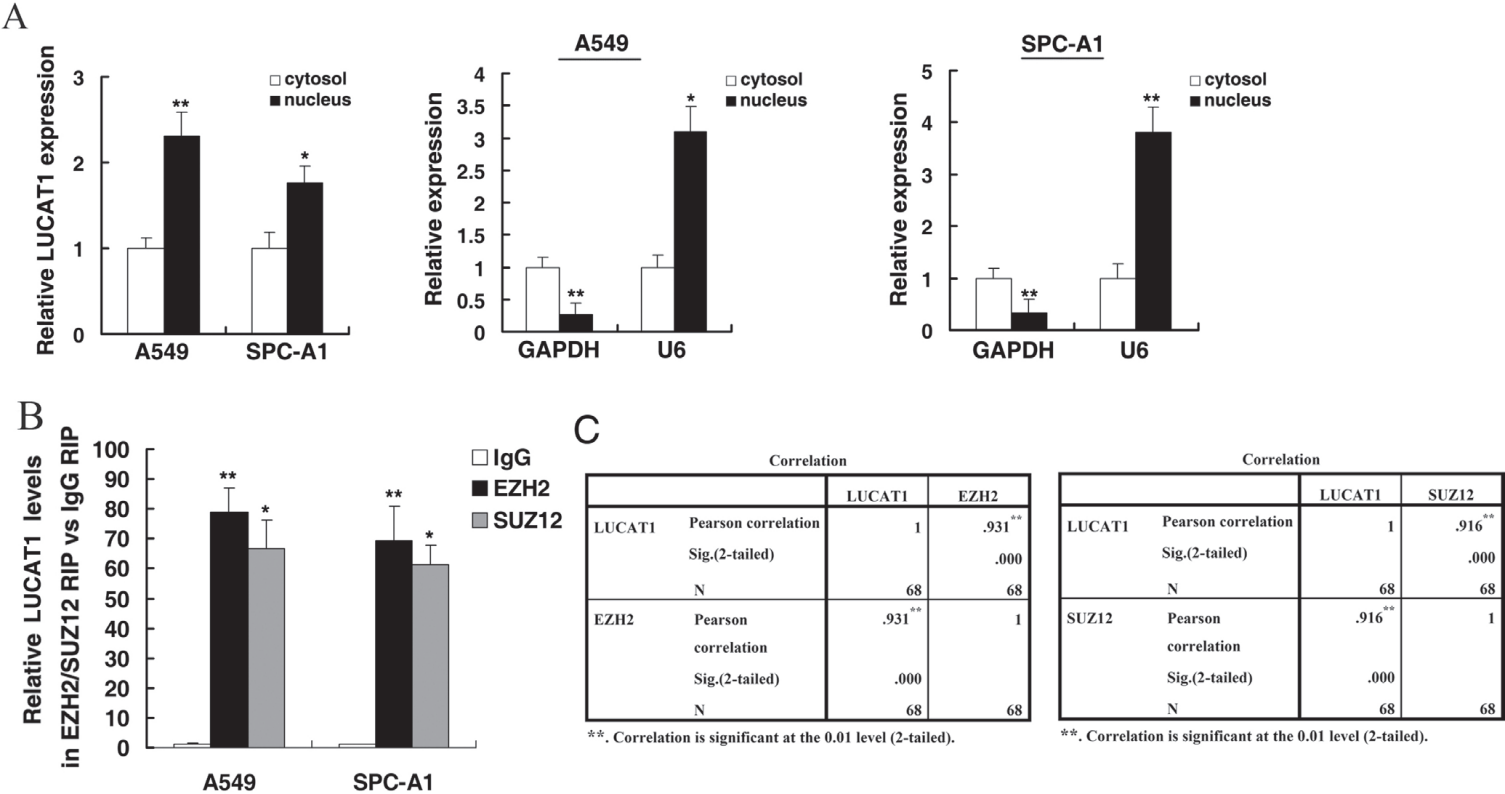
Subcellular fractionation location of LUCAT1, and LUCAT1 could bind to PRC2 (**A**) LUCAT1 nuclear localization, as identified using qRT-PCR in fractionated A549 and SPC-A1 cells. After nuclear and cytosolic separation, RNA expression was measured by qRT-PCR. GAPDH was used as a cytosolic marker, and U6 was used as a nuclear marker. (**B**) RIP experiments were performed in A549 and SPC-A1 cells and the coprecipitated RNA were subjected to qRT-PCR for LUCAT1. The fold enrichment of LUCAT1 in EZH2/SUZ12 RIP is relative to its matching IgG control RIP. (**C**) The level of LUCAT1 expression in NSCLC tissues showed a statistically positive correlation with the relative level of EZH2 and SUZ12 expression (*N* = 68). *P < 0.05, **P < 0.01.

Khalil et al. demonstrated that nearly twenty percent of all human lncRNAs physically associate with PRC2 [22]. Furthermore, recent studies have reported that the expression of EZH2, a catalytic subunit of PRC2, is up-regulated in several advanced cancers, such as lung cancer [18], breast cancer [23], and prostate cancer [24]. Therefore, we hypothesized that LUCAT1 could regulate gene expression by binding to PRC2. First, we performed qRT-PCR to measure LUCAT1 expression in nuclear and cytosolic fractions, using GAPDH as a cytosol marker and U6 as a nucleus marker. As shown in Figure 6A, we found that LUCAT1 expression was increased markedly in the nucleus compared to the cytosol, suggesting that LUCAT1 might play a role in transcriptional regulation.

To further explore the functional role of LUCAT1 in NSCLC cell proliferation, we conducted RIP analysis to test whether LUCAT1 could bind PRC2 in NSCLC cells. The results revealed that endogenous LUCAT1 was enriched, relative to the input, in the anti-EZH2/SUZ12 RIP fraction compared with the IgG fraction both in A549 and SPC-A1cell lines (Figure 6B). Moreover, we detected expression of EZH2/SUZ12 by qPCR and found that LUCAT1 were positively correlated with EZH2/SUZ12 expression in 68 pairs NSCLC tissues (Figure 6C).

### LUCAT1 functions as a tumor activator by epigenetically regulating p21 and p57 expression which are required to target EZH2 occupancy

**Figure 7:**
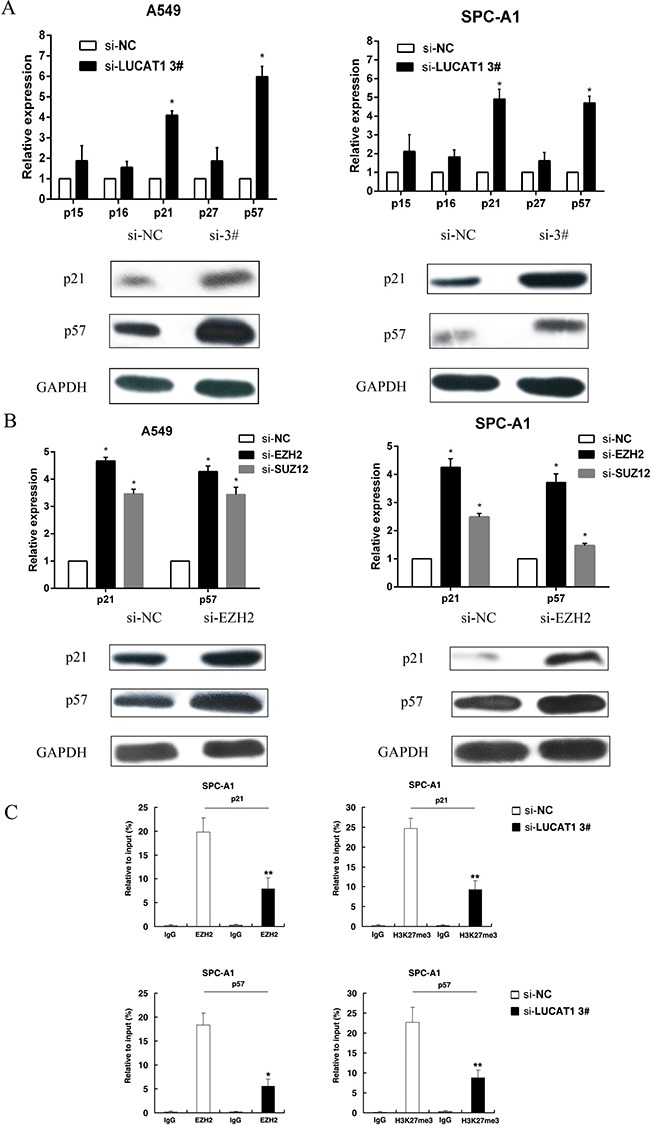
LUCAT1 is required to target EZH2 occupancy and activity to epigenetically regulate the expression of CKIs (**A**) After si-LUCAT1 3# transfection, the expression of p15, p16, p21, p27, and p57 were determined by qPCR and western blot assays in A549 and SPC-A1 cells. (**B**) After si-EZH2 or si-SUZ12 transfection, the expression of p21 and p57 were detected by qPCR in A549 and SPC-A1 cells. And western blot assay was performed to detect the protein level of p21 and p57 after si-EZH2 transfection. (**C**) ChIP-qPCR of H3K27me3 and EZH2 of the promoter region of the p21 and p57 locus after siRNA treatment targeting si-NC or si-LUCAT1 3# in SPC-A1 cells. Antibody enrichment was quantified relative to input controls. Antibody directed against IgG was used as a negative control. Error bars indicate means ± S.E.M. *P < 0.05, **P < 0.01.

To further study the effect of LUCAT1 in G0/G1 arrest, we performed qRT-PCR to evaluate the role of LUCAT1 inhibition on the expression of cyclin-dependent protein kinase inhibitors (CKIs), whose deregulation leads to uncontrolled proliferation and tumorigenesis [25]. The results showed that p21 and p57 were increased with knockdown of LUCAT1 (Figure 7A). Then we evaluated the protein levels of p21 and p57 by western blotting and showed that the levels of both proteins were increased with LUCAT1 knockdown. Taken together, these data indicate that LUCAT1 might lead to cell-cycle arrest by down-regulating the expression of the tumor suppressors p21 and p57. We also asked whether EZH2 was involved in the inhibition of p21 and p57 by evaluating the expression of p21 and p57 by qRT-PCR and western blotting after EZH2 knockdown. As shown in Figure 7B, the expression of the tumor suppressors p21 and p57 was increased in both A549 and SPC-A1 cell lines. To avoid off-target effects and ensure the efficiency of RNA interference, we used an interference target sequence against EZH2 that had been validated in previous work [26].

To explore the mechanism by which LUCAT1 regulates p21 and p57, a ChIP assay was performed. We conducted ChIP analysis in the SPC-A1 cell line after LUCAT1-knockdown, and the results showed that knockdown of LUCAT1 decreased the binding of EZH2 and dimethylation of lysine 27 on histone 3(H3K27me3) levels across the p21 and p57 promoters compared to the control group (Figure 7C). These results suggest that LUCAT1 promotes NSCLC cell proliferation through epigenetically silencing p21 and p57 expression, which is required to target EZH2 occupancy.

## DISCUSSION

Recently, considerable research effort has been dedicated to elucidating the biological functions and clinical impact of long non-coding RNAs. One important finding from these studies is that lncRNAs might be novel biomarkers for several types of human cancers, including NSCLC [27, 28]. For example, the down-regulated expression of lncRNA HMlincRNa717 was associated with poor overall survival of NSCLC patients [29]. Lin et al. reported that lncRNA ANRIL could be a potential biomarker for NSCLC prognosis and might have a functional role in NSCLC progression [30]. M. Sun et al. found that down-regulation of lncRNA BRAF suppressed NSCLC metastasis, induced cell apoptosis, and was associated with the prognosis of NSCLC patients [31]. E.B. Zhang et al. demonstrated that lncRNA TUG1 is regulated by P53-dependent cell proliferation in NSCLC via epigenetic regulation of HOXB7 expression [32]. However, the functional role and clinicopathologic significance of lncRNA LUCAT1 in human NSCLC remain unclear.

Tumor growth is the process that how the initiating cells evolve into a visible tumor mass, and it includes cancer cell proliferation, preventing cell death, and angiogenesis [33]. Numerous studies have found that many lncRNAs could promote or inhibit cell proliferation, and their abnormal expression could lead to cancer cell growth. For example, PCGEM1 is highly up-regulated in prostate cancers, and it could promote prostate cancer cell proliferation [34]. Conversely, GAS5 could control cell growth and is down-regulated in breast cancer [10]. In addition, aberrant expression of lncRNAs is involved in diverse tumors and can function as prognostic indicators [9, 25]. In our present study, we found that lncRNA LUCAT1 expression was up-regulated in NSCLC tissues. We also demonstrated that LUCAT1 knockdown led to a significant suppression of cell proliferation both *in vitro* and *in vivo*, induced G0/G1 arrest, and promoted cell apoptosis. Moreover, we showed that NSCLC patients with elevated expression of LUCAT1 had a shorter overall survival than those with low expression, suggesting that lncRNA LUCAT1 has a high prognostic value in NSCLC. These findings suggest that lncRNA LUCAT1 may play an important role in NSCLC growth and could be an independent prognostic indicator or progression marker for NSCLC patients.

PRC2 is composed of EZH2, SUZ12, and EED. And it can medicate the marks which is associated with genetic silence, such as dimethylation of lysine 9 (H3K9me2) and H3K27me3 [35]. What's more, considerable evidence has certified that PRC2 is interacted with many lncRNAs, one example is that the HOX transcript antisense RNA (HOTAIR) can function with PRC2 together to regulate the inhibition of the homeobox D cluster (HOXD) locus via spreading H3K27me3 marks, which are correlated with gene silencing [36]. Furthermore, previous studies have shown that lncRNA can influence tumor biology via binding to EZH2 in various cancers. And EZH2 can be regarded as a suppressor of cancer progression [16, 37]. Thus, we hypothesized that LUCAT1 might affect gene expression in a similar manner. We performed RIP and the results revealed that LUCAT1 could recruit and bind to EZH2. It is evident that LUCAT1 can participate in PRC2-mediated epigenetic regulation and may play an important role in the occurrence and progression of NSCLC.

CKIs are categorized into two families: INK4 family and Cip/Kip family. It is well known that CKIs can govern cell cycle progression by negatively regulating cyclin-dependent kinases (CDKs) [38]. In addition, researchers have reported that PRC2-mediated histone methylation has an effect on the inhibition of CKIs [39–41]. In our study, we found that the cell-cycle progression of si-LUCAT1 cells was significantly arrested in G0/G1 phase. Therefore, we explored the effect of LUCAT1 in G0/G1 arrest by evaluating the role of LUCAT1 inhibition on the expression of CKIs. The results showed that the expression of p21 and p57 clearly increased as a result of LUCAT1 knockdown. Previous studies have revealed that promoter hypermethylation of p21 is present in 30% of NSCLCs [42]. Additionally, the inactivation of the p57 gene resulting from promoter DNA methylation was observed in NSCLC and in lymphoid malignancies of B-cells [43, 44]. Furthermore, p21 and p57 expression have shown to be reduced in a variety of cancers, including hepatocellular carcinoma, colorectal carcinoma and breast cancer [45–48]. We then employed a ChIP assay and our data revealed that knockdown of LUCAT1 decreased both the binding of EZH2 and H3K27me3 levels across the p21 and p57 promoters compared to the control group (Figure 6C). This suggested that LUCAT1 could down-regulate p21 and p57 levels and was required for EZH2 binding to p21 and p57 promoters. Taken together, we concluded that LUCAT1 might play a vital role in the cell cycle of NSCLC via epigenetically silencing p21 and p57 expression.

**Figure 8:**
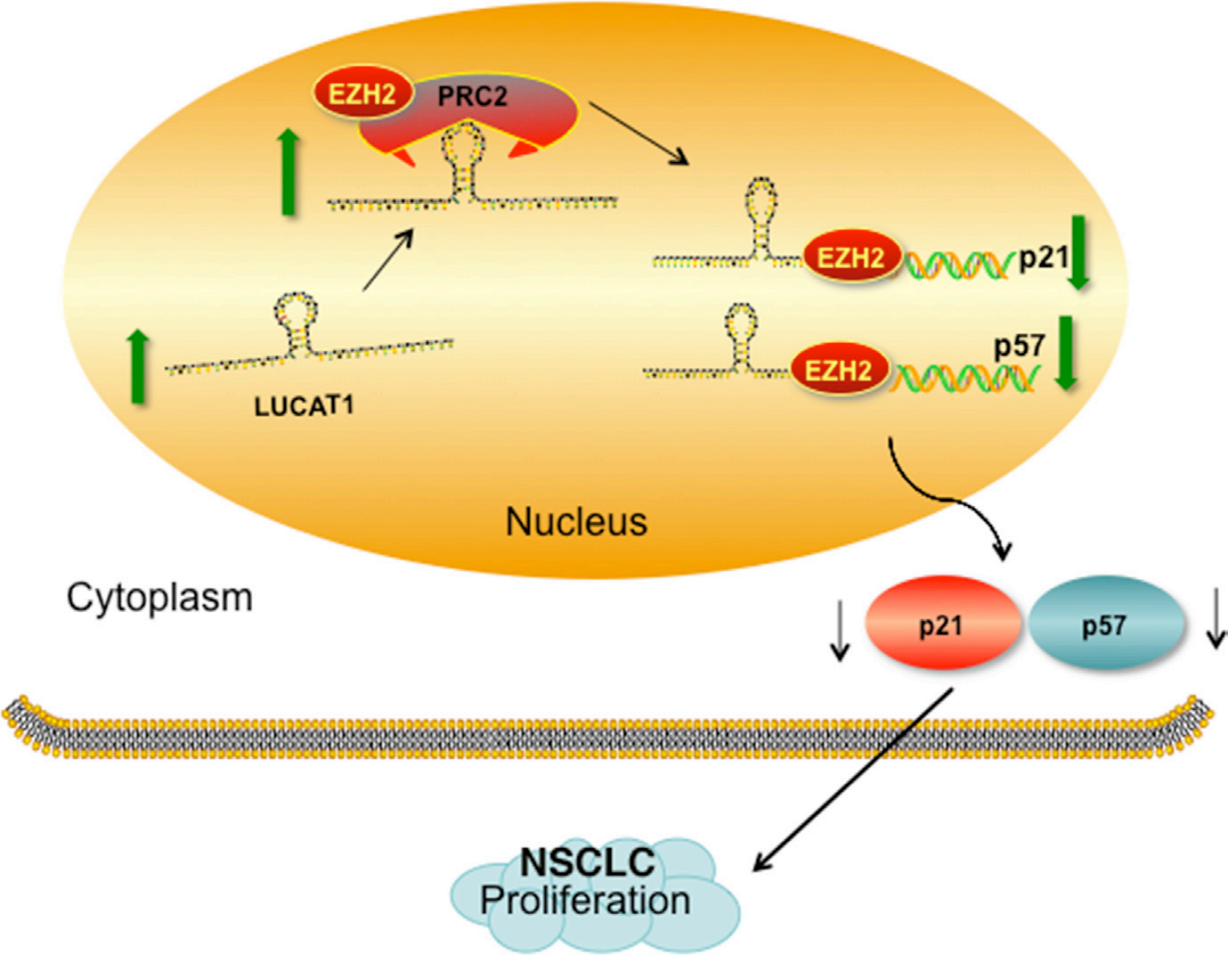
Proposed model which medicated by LUCAT1 in proliferation of NSCLC

In summary, our study reveals that lncRNA LUCAT1 expression is significantly up-regulated in NSCLC tissues and cell lines. Its abnormal expression can predict a poor prognosis of NSCLC patients, and it may be an independent prognostic marker. Knockdown of LUCAT1 inhibited cell proliferation both *in vitro* and *in vivo*. LUCAT1 mediated the oncogenic effects is partially through its epigenetic silencing of the p21 and p57 expression by binding to PRC2 (Figure 8). Our findings provide a new potential target and therapeutic strategy for patients with NSCLC. Nevertheless, additional possible molecular mechanisms by which LUCAT1 participates in NSCLC cell biological function requires further investigation.

## MATERIALS AND METHODS

### Patients and sample collection

Human NSCLC tissues and corresponding non-tumor tissues were obtained from patients undergoing surgical resection at the First Affiliated Hospital of Nanjing Medical University. All tissue samples were immediately snap-frozen in liquid nitrogen after surgical resection. None of the patients received any cancer treatment before surgical treatment. This project was approved by the Research Ethics Committee of Nanjing Medical University (Nanjing, Jiangsu, PR China), and written informed consent was obtained from all patients. The clinicopathological characteristics of the NSCLC cancer patients are summarized in Table 3.

**Table 3:** The clinic-pathological factors of NSCLC patients

Clinical factors	Number of cases	(%) of patients
**Sex**		
male	44	64.7
female	24	35.3
**Age**		
≤ 60	37	54.4
> 60	31	45.6
**Histological grade**		
high	9	13.2
middle	22	32.4
middle to low	17	25.0
low	19	27.9
other	1	1.5
**Histological classification**		
SCC (Squamous cell carcinoma)	40	58.8
AD(adenocarcinoma)	24	35.3
other	4	5.9
**Tumor stage**		
I	18	26.5
II	31	45.6
III	17	25.0
IV	2	2.9
**Tumor (T)**		
T1	20	29.4
T2	30	44.1
T3	13	19.1
T4	5	7.4
**Lymph node metastasis (N)**		
N0	29	42.6
N1	31	45.6
N2	7	10.3
N3	1	1.5
**History of smoking**		
Ever	42	61.8
never	26	38.2

### Lung cancer RNA-expression profiling data retrieval and analysis

RNA-Seq data (from TCGA: The Cancer Genome Atlas) of lncRNAs of lung adenocarcinoma and lung squamous cell carcinoma were analyzed by Bioinformatics Tools “lncRNAtor” (http://lncrnator.ewha.ac.kr/index.htm).

### Cell lines and cultures

The human NSCLC cell lines (A549, SPC-A1 and H1703) and the normal bronchial epithelial cell line (HBE) were purchased from the Institute of Biochemistry and Cell Biology of the Chinese Academy of Sciences (Shanghai, China). The cells were cultured in RPMI 1640 or DMEM (GIBCO-BRL) medium which was supplemented with 10% fetal bovine serum (10% FBS), 100 U/ml penicillin, and 100 mg/ml streptomycin in a humidified incubator at 37°C with 5% CO2.

### Cell transfection

Non-small lung cancer cells were transfected with siRNA and plasmid vectors using Lipofectamine 2000 (Invitrogen, USA) according to the manufacturer's instructions. Three individual LUCAT1 siRNAs (si-LUCAT1 1#, 2#, and 3#) and negative control siRNA (si-NC) were purchased from Invitrogen (Invitrogen, USA). The nucleotide sequences of the siRNA for LUCAT1 are listed in Supplementary Table 1. At 48 h post-transfection, cells were harvested for further studies. LUCAT1 cDNA was synthesized and cloned into the expression vector pcDNA3.1 (Invitrogen).

### RNA extraction and quantitative real-time PCR (qRT-PCR)

Total RNA was extracted from tissue samples and cultured cells using TRIzol reagent (Invitrogen, Carlsbad, CA) according to the manufacturer's instructions. RNA was reverse transcribed to cDNA by using a Reverse Transcription Kit (Takara, Dalian, China) for qRT-PCR. The qRT-PCR was performed to detect the expression of LUCAT1 in NSCLC and non-malignant tissues by using SYBR Green (Takara, Dalian China), with GAPDH as a normalizing control. The primers for lncRNA LUCAT1 are listed in Supplementary Table 1. The relative expression of lncRNA LUCAT1 was calculated by the double-standard curves method after normalizing to GAPDH expression.

### Cell proliferation assays

Cell proliferation was detected using the Cell Proliferation Reagent Kit I (MTT) (Roche, Basel, Switzerland). In brief, the transfected cells were seeded at a density of 2 ×10^3^ cells per well in 200 μl culture medium in 96-well plates. Cell proliferation was assessed every 24 h following the manufacturer's protocol. For the colony formation assay, specific numbers of transfected cells were placed into each well of a six-well plate and cultured in medium containing 10% FBS for 15 days, replacing the medium every 5 days. Colonies were fixed with methanol and stained with 0.1% crystal violet (Sigma-Aldrich, St. Louis, MO, USA) in PBS for 15 min. Colony formation was determined by counting the number of stained colonies. For each treatment group, wells were counted in triplicate.

### Western blot assay and antibodies

Cells were collected after 48 hours of transfection and lysed using RIPA protein extraction reagent (Beyotime, Beijing, China) supplemented with a protease inhibitor cocktail (Roche, CA, USA) and phenylmethylsulfonyl fluoride (Roche). Cell protein lysates were separated by 10% SDS-polyacrylamide gel electrophoresis (SDS-PAGE) and transferred onto 0.22-μm NC membranes (Sigma). The membranes were incubated with specific antibodies. Autoradiograms were quantified by densitometry (Quantity One software; Bio-Rad), and GAPDH was used as a control. The specific antibodies for GAPDH, p21 and p57 were purchased from Cell Signaling Technology, Inc. Antibodies against EZH2 were purchased from Abcam.

### Flow cytometry analysis of apoptosis and cell cycle

Transfected A549 and SPCA1 cells were seeded in six-well culture dishes for 48 h. For cell cycle analysis, cells were stained with propidium iodide using the CycleTEST PLUS DNA Reagent Kit (BD Biosciences) according to the manufacturer's instructions and analyzed by FACScan. The percentages of cells in G0/G1, S, and G2/M phase were counted and compared. Adherent cells and suspended cells were collected for apoptosis analysis. After staining with FITCAnnexin V and propidium iodide, cells were analyzed by FACScan using CellQuest software (BD Biosciences). Cells were discriminated into viable cells, dead cells, early apoptotic cells and apoptotic cells. The relative ratio of early apoptotic cells were compared with control transfection from each experiment. The experiment was performed independently three times for each cell line.

### Tumor formation assay in a nude mouse model

Animal care and protocols were approved by the Shanghai Medical Experimental Animal Care Commission. Five-week-old athymic BALB/c mice were maintained under specific pathogen-free conditions. SPCA1 cells stably transfected with Scramble or shLUCAT1 were harvested at a concentration of 2×10^7^ cells/ml. Of the suspending cells, 0.1 ml was subcutaneously injected into the flanks of the nude mouse, one injection per mouse. The volumes of the tumor were measured every 2 days when the implantations started to grow. Three weeks after injection, the mice were killed, and the tumors were resected, measured, and weighed. Tumor volume was calculated using the equation: volume = 0.5 × W^2^ × L (W, width; L, length). Then, the tumor tissues were fixed in 10% formalin solution for immunostaining analysis.

### Immunohistochemistry

The primary tumors were immunostained as previously described [49].

### Subcellular fractionation

The separation of nuclear and cytosolic fractions was performed using the PARIS Kit (Life Technologies, Carlsbad, CA, USA) according to the manufacturer's instructions.

### RNA-binding protein immunoprecipitation (RIP) assay

RIP experiments were performed using a Magna RIP™ RNA-Binding Protein Immunoprecipitation Kit (Millipore, USA) following the manufacturer's protocol. Cells were lysed and 100μl of whole cell extract was incubated with RIP buffer containing magnetic beads conjugated with antibodies that recognized EZH2, SUZ12 or with control IgG (millipore) for 6 h at 4°C. Antibodies for RIP assays against EZH2 and SUZ12 were purchased from Abcam. After washing the beadswith wash buffer, the complexes were incubated with 0.1% SDS/0.5 mg/ml Proteinase K (30 min at 55°C) to remove proteins. The RNA concentration was measured using a NanoDrop (Thermo Scientific), and the RNA quality assessed using a bioanalyser (Agilent, Santa Clara, CA, USA). After the immunoprecipitated RNA was isolated and purified, qRT-PCR was performed.

### Chromatin immunoprecipitation (ChIP) assays

Simple ChIP Enzymatic Chromatin IP Kit (Millipore, USA) was used for ChIP assays according to the manufacturer's protocol. Cells were cross-linked with formaldehyde and collected in lysis buffer. Cell lysates were then sonicated to generate chromatin fragments of 200–300 bp and immunoprecipitated with EZH2 and H3K27me3-specific antibodies (CST) or IgG as control. Immunoprecipitated DNA was amplified by PCR using primers, which are listed in Supplementary Table 1. EZH2 antibodies were obtained from Abcam. The H3 trimethyl Lys 27 antibody was purchased from Millipore. Precipitated chromatin DNA was recovered and analyzed by qRT-PCR.

### Statistical analysis

Statistical analyses were performed using SPSS 20.0 software (IBM, SPSS, USA). The experimental results were statistically analyzed for significant differences using Student's *t*-test or the chi-square test. Overall survival curves were plotted following the Kaplan-Meier method and were analyzed with the log-rank test. Univariate and multivariate Cox proportional hazards models were applied to analyze the survival variables. A *P* < 0.05 was considered statistically significant.

## SUPPLEMENTARY MATERIALS TABLE



## References

[1] Goldstraw P, Crowley J, Chansky K, Giroux DJ, Groome PA, Rami-Porta R, Postmus PE, Rusch V, Sobin L (2007). The IASLC Lung Cancer Staging Project: proposals for the revision of the TNM stage groupings in the forthcoming (seventh) edition of the TNM Classification of malignant tumours. Journal of thoracic oncology.

[2] DeSantis CE, Lin CC, Mariotto AB, Siegel RL, Stein KD, Kramer JL, Alteri R, Robbins AS, Jemal A (2014). Cancer treatment and survivorship statistics, 2014. CA Cancer J Clin.

[3] Travis WD (2011). Pathology of lung cancer. Clinics in chest medicine.

[4] Tzankov A, Gschwendtner A, Augustin F, Fiegl M, Obermann EC, Dirnhofer S, Went P (2006). Diffuse large B-cell lymphoma with overexpression of cyclin e substantiates poor standard treatment response and inferior outcome. Clinical cancer research.

[5] Amaral PP, Dinger ME, Mercer TR, Mattick JS (2008). The eukaryotic genome as an RNA machine. Science.

[6] Guttman M, Amit I, Garber M, French C, Lin MF, Feldser D, Huarte M, Zuk O, Carey BW, Cassady JP, Cabili MN, Jaenisch R, Mikkelsen TS (2009). Chromatin signature reveals over a thousand highly conserved large non-coding RNAs in mammals. Nature.

[7] Hauptman N, Glavac D (2013). Long non-coding RNA in cancer. International journal of molecular sciences.

[8] Gutschner T, Diederichs S (2012). The hallmarks of cancer: a long non-coding RNA point of view. RNA biology.

[9] Gutschner T, Hammerle M, Eissmann M, Hsu J, Kim Y, Hung G, Revenko A, Arun G, Stentrup M, Gross M, Zornig M, MacLeod AR, Spector DL (2013). The noncoding RNA MALAT1 is a critical regulator of the metastasis phenotype of lung cancer cells. Cancer research.

[10] Mourtada-Maarabouni M, Pickard MR, Hedge VL, Farzaneh F, Williams GT (2009). GAS5, a non-protein-coding RNA, controls apoptosis and is downregulated in breast cancer. Oncogene.

[11] Schmidt LH, Spieker T, Koschmieder S, Schaffers S, Humberg J, Jungen D, Bulk E, Hascher A, Wittmer D, Marra A, Hillejan L, Wiebe K, Berdel WE (2011). The long noncoding MALAT-1 RNA indicates a poor prognosis in non-small cell lung cancer and induces migration and tumor growth. Journal of thoracic oncology.

[12] Yu H, Xu Q, Liu F, Ye X, Wang J, Meng X (2015). Identification and validation of long noncoding RNA biomarkers in human non-small-cell lung carcinomas. Journal of thoracic oncology.

[13] Yang J, Lin J, Liu T, Chen T, Pan S, Huang W, Li S (2014). Analysis of lncRNA expression profiles in non-small cell lung cancers (NSCLC) and their clinical subtypes. Lung cancer.

[14] Brockdorff N (2013). Noncoding RNA and Polycomb recruitment. RNA.

[15] Yoo KH, Hennighausen L (2012). EZH2 methyltransferase and H3K27 methylation in breast cancer. International journal of biological sciences.

[16] Chase A, Cross NC (2011). Aberrations of EZH2 in cancer. Clinical cancer research.

[17] Matsukawa Y, Semba S, Kato H, Ito A, Yanagihara K, Yokozaki H (2006). Expression of the enhancer of zeste homolog 2 is correlated with poor prognosis in human gastric cancer. Cancer science.

[18] Watanabe H, Soejima K, Yasuda H, Kawada I, Nakachi I, Yoda S, Naoki K, Ishizaka A (2008). Deregulation of histone lysine methyltransferases contributes to oncogenic transformation of human bronchoepithelial cells. Cancer cell international.

[19] Liu Y, Liu T, Bao X, He M, Li L, Yang X (2014). Increased EZH2 expression is associated with proliferation and progression of cervical cancer and indicates a poor prognosis. International journal of gynecological pathology.

[20] Sudo T, Utsunomiya T, Mimori K, Nagahara H, Ogawa K, Inoue H, Wakiyama S, Fujita H, Shirouzu K, Mori M (2005). Clinicopathological significance of EZH2 mRNA expression in patients with hepatocellular carcinoma. British journal of cancer.

[21] Thai P, Statt S, Chen CH, Liang E, Campbell C, Wu R (2013). Characterization of a novel long noncoding RNA, SCAL1, induced by cigarette smoke and elevated in lung cancer cell lines. American journal of respiratory cell and molecular biology.

[22] Khalil AM, Guttman M, Huarte M, Garber M, Raj A, Rivea Morales D, Thomas K, Presser A, Bernstein BE, van Oudenaarden A, Regev A, Lander ES, Rinn JL (2009). Many human large intergenic noncoding RNAs associate with chromatin-modifying complexes and affect gene expression. Proceedings of the National Academy of Sciences of the United States of America.

[23] Raaphorst FM, Meijer CJ, Fieret E, Blokzijl T, Mommers E, Buerger H, Packeisen J, Sewalt RA, Otte AP, van Diest PJ (2003). Poorly differentiated breast carcinoma is associated with increased expression of the human polycomb group EZH2 gene. Neoplasia.

[24] Foster CS, Falconer A, Dodson AR, Norman AR, Dennis N, Fletcher A, Southgate C, Dowe A, Dearnaley D, Jhavar S, Eeles R, Feber A, Cooper CS (2004). Transcription factor E2F3 overexpressed in prostate cancer independently predicts clinical outcome. Oncogene.

[25] Cicenas J, Valius M (2011). The CDK inhibitors in cancer research and therapy. Journal of cancer research and clinical oncology.

[26] Prensner JR, Iyer MK, Balbin OA, Dhanasekaran SM, Cao Q, Brenner JC, Laxman B, Asangani IA, Grasso CS, Kominsky HD, Cao X, Jing X, Wang X (2011). Transcriptome sequencing across a prostate cancer cohort identifies PCAT-1, an unannotated lincRNA implicated in disease progression. Nature biotechnology.

[27] Lee JT (2012). Epigenetic regulation by long noncoding RNAs. Science.

[28] Prensner JR, Chinnaiyan AM (2011). The emergence of lncRNAs in cancer biology. Cancer discovery.

[29] Xie X, Liu HT, Mei J, Ding FB, Xiao HB, Hu FQ, Hu R, Wang MS (2014). LncRNA HMlincRNA717 is down-regulated in non-small cell lung cancer and associated with poor prognosis. International journal of clinical and experimental pathology.

[30] Lin L, Gu ZT, Chen WH, Cao KJ Increased expression of the long non-coding RNA ANRIL promotes lung cancer cell metastasis and correlates with poor prognosis. Diagnostic pathology.

[31] Sun M, Liu XH, Wang KM, Nie FQ, Kong R, Yang JS, Xia R, Xu TP, Jin FY, Liu ZJ, Chen JF, Zhang EB, De W Downregulation of BRAF activated non-coding RNA is associated with poor prognosis for non-small cell lung cancer and promotes metastasis by affecting epithelial-mesenchymal transition. Molecular cancer.

[32] Zhang EB, Yin DD, Sun M, Kong R, Liu XH, You LH, Han L, Xia R, Wang KM, Yang JS, De W Shu YQ, Wang ZX P53-regulated long non-coding RNA TUG1 affects cell proliferation in human non-small cell lung cancer, partly through epigenetically regulating HOXB7 expression. Cell death & disease.

[33] Yang G, Lu X, Yuan L (2014). LncRNA: a link between RNA and cancer. Biochimica et biophysica acta.

[34] Fu X, Ravindranath L, Tran N, Petrovics G, Srivastava S (2006). Regulation of apoptosis by a prostate-specific and prostate cancer-associated noncoding gene, PCGEM1. DNA and cell biology.

[35] Czermin B, Melfi R, McCabe D, Seitz V, Imhof A, Pirrotta V (2002). Drosophila enhancer of Zeste/ESC complexes have a histone H3 methyltransferase activity that marks chromosomal Polycomb sites. Cell.

[36] Rinn JL, Kertesz M, Wang JK, Squazzo SL, Xu X, Brugmann SA, Goodnough LH, Helms JA, Farnham PJ, Segal E, Chang HY (2007). Functional demarcation of active and silent chromatin domains in human HOX loci by noncoding RNAs. Cell.

[37] Benetatos L, Voulgaris E, Vartholomatos G, Hatzimichael E (2013). Non-coding RNAs and EZH2 interactions in cancer: long and short tales from the transcriptome. International journal of cancer.

[38] Lim S, Kaldis P (2013). Cdks, cyclins and CKIs: roles beyond cell cycle regulation. Development.

[39] Paul TA, Bies J, Small D, Wolff L (2010). Signatures of polycomb repression and reduced H3K4 trimethylation are associated with p15INK4b DNA methylation in AML. Blood.

[40] Fan T, Jiang S, Chung N, Alikhan A, Ni C, Lee CC, Hornyak TJ (2011). EZH2-dependent suppression of a cellular senescence phenotype in melanoma cells by inhibition of p21/CDKN1A expression. Molecular cancer research.

[41] Yang X, Karuturi RK, Sun F, Aau M, Yu K, Shao R, Miller LD, Tan PB, Yu Q CDKN1C (p57) is a direct target of EZH2 and suppressed by multiple epigenetic mechanisms in breast cancer cells. PloS one.

[42] Teramen H, Tsukuda K, Tanaka N, Ueno T, Kubo T, Ando M, Soh J, Asano H, Pass HI, Toyooka S, Miyoshi S (2011). Aberrant methylation of p21 gene in lung cancer and malignant pleural mesothelioma. Acta medica Okayama.

[43] Li Y, Nagai H, Ohno T, Yuge M, Hatano S, Ito E, Mori N, Saito H, Kinoshita T (2002). Aberrant DNA methylation of p57(KIP2) gene in the promoter region in lymphoid malignancies of B-cell phenotype. Blood.

[44] Hagiwara K, Li Y, Kinoshita T, Kunishma S, Ohashi H, Hotta T, Nagai H (2010). Aberrant DNA methylation of the p57KIP2 gene is a sensitive biomarker for detecting minimal residual disease in diffuse large B cell lymphoma. Leukemia research.

[45] Abbas T, Dutta A (2009). p21 in cancer: intricate networks and multiple activities. Nature reviews Cancer.

[46] Hu T, Guo H, Wang W, Yu S, Han L, Jiang L, Ma J, Yang C, Guo Q, Nan K (2013). Loss of p57 expression and RhoA overexpression are associated with poor survival of patients with hepatocellular carcinoma. Oncology reports.

[47] Li JQ, Wu F, Usuki H, Kubo A, Masaki T, Fujita J, Bandoh S, Saoo K, Takeuchi H, Kuriyama S, Ishida T, Imaida K (2003). Loss of p57KIP2 is associated with colorectal carcinogenesis. International journal of oncology.

[48] Xu XY, Wang WQ, Zhang L, Li YM, Tang M, Jiang N, Cai SL, Wei L, Jin F, Chen B (2012). Clinical implications of p57 KIP2 expression in breast cancer. Asian Pacific journal of cancer prevention.

[49] Liao WT, Wang X, Xu LH, Kong QL, Yu CP, Li MZ, Shi L, Zeng MS, Song LB (2009). Centromere protein H is a novel prognostic marker for human nonsmall cell lung cancer progression and overall patient survival. Cancer.

