# Supplementary material for: Long non-coding RNA LUCAT1 is associated with poor prognosis in human non-small cell lung cancer and regulates cell proliferation via epigenetically repressing p21 and p57 expression
Source: Oncotarget. 2017 Mar 9;8(17):28297–311. doi: 10.18632/oncotarget.16044 (PMC5438651; doi:10.18632/oncotarget.16044)
Supplement: Supplementary file 1 [file oncotarget-08-28297-s001.pdf]

Long non-coding RNA LUCAT1 is associated with poor prognosis in human non-small lung cancer and regulates cell proliferation via epigenetically repressing p21 and p57 expression

Supplementary Materials

Supplementary Table 1: The list of the nucleotide sequences and primers

The list of the nucleotide	sequences of the siRNA for LUCAT1	
**si-LUCAT1**	**The nucleotide sequences**	
si-RNA 1#	sense 5′-CCCAUCAGAAGAUGUCAGAAGAUAA-3′	
	antisense 5′-UUAUCUUCUGACAUCUUCUGAUGGG-3′	
si-RNA 2#	sense 5′-UUAAGAAGUAGAACACUGAGGGACA-3′	
	antisense 5′-UGUCCCUCAGUGUUCUACUUCUUAA-3′	
si-RNA 3#	sense 5′-CAAGCUCUUGCAGUCAACAAGAACU-3′	
	antisense 5′-AGUUCUUGUUGACUGCAAGAGCUUG-3′	
**The list of primers**		
**Primer name**	F(5′—3′)	R(5′—3′)
**LUCAT1**	ACCAGCTGTCCCTCAGTGTTCT	AGGCCTTTATCCTCGGGTTGCCT
**p15**	GGACTAGTGGAGAAGGTGCG	GGGCGCTGCCCATCATCATG
**p16**	CACCGAATAGTTACGGTCGG	GCACGGGTCGGGTGAGAGTG
**p21**	GTCCACTGGGCCGAAGAG	TGCGTTCACAGGTGTTTCTG
**p27**	TGCAACCGACGATTCTTCTACTCAA	CAAGCAGTGATGTATCTGATAAACAAGG
**p57**	CTAGCCAGCAGGCATCGAG	GTGGTGGACTCTTCTGCGTC
**The list of primers for ChIP**	assays	
**ChIP Primers**	F(5′—3′)	R(5′—3′)
**p21**	GCCTTCCTCACATCCTCC	CAAGAGTGCCCAGTCCAG
**p57**	TCCATCTACTGGGCAGGGTG	ACAGGGGTCAGCTCCACTCT
**shLUCAT1**	Sequences cloned into pRNAT-H1.1/NEO vector	
	F	
	GATCCCAAGCUCUUGCAGUCAACAAGAA CUUCAAGAGAAGUUCUUGUUGACUGCAAGAGCUUGTTTTTA	
	R	
	AGCTTAAAAAACAAGCUCUUGCAGUCAA CAAGAACUCUCTTGAAAGUUCUUGUUGACUGCAAGAGCUUGG	
